# Modified Delphi Consensus to Suggest Key Elements of *Stepping On* Falls Prevention Program

**DOI:** 10.3389/fpubh.2017.00021

**Published:** 2017-02-20

**Authors:** Jane E. Mahoney, Lindy Clemson, Amy Schlotthauer, Karin A. Mack, Terry Shea, Vicki Gobel, Sandy Cech

**Affiliations:** ^1^Department of Medicine, University of Wisconsin School of Medicine and Public Health, Madison, WI, USA; ^2^The University of Sydney, Ageing and Occupational Therapy, Sydney, NSW, Australia; ^3^Injury Research Center, Medical College of Wisconsin, Milwaukee, WI, USA; ^4^Centers for Disease Control and Prevention, National Center for Injury Prevention and Control, Atlanta, GA, USA; ^5^Greater Wisconsin Agency on Aging Resources, Inc., Madison, WI, USA

**Keywords:** fall prevention, implementation, health promotion, Delphi consensus, *Stepping On*

## Abstract

Falls among older adults result in substantial morbidity and mortality. Community-based programs have been shown to decrease the rate of falls. In 2007, the Centers for Disease Control and Prevention funded a research study to determine how to successfully disseminate the evidence-based fall prevention program (*Stepping On*) in the community setting. As the first step for this study, a panel of subject matter experts was convened to suggest which parts of the *Stepping On* fall prevention program were considered key elements, which could not be modified by implementers.

**Methods:**

Older adult fall prevention experts from the US, Canada, and Australia participated in a modified Delphi technique process to suggest key program elements of *Stepping On*. Forty-four experts were invited to ensure that the panel of experts would consist of equal numbers of physical therapists, occupational therapists, geriatricians, exercise scientists, and public health researchers. Consensus was determined by percent of agreement among panelists. A Rasch analysis of item fit was conducted to explore the degree of diversity and/or homogeneity of responses across our panelists.

**Results:**

The Rasch analysis of the 19 panelists using fit statistics shows there was a reasonable and sufficient range of diverse perspectives (Infit MnSQ 1.01, *Z* score −0.1, Outfit MnSQ 0.96, *Z* score −0.2 with a separation of 4.89). Consensus was achieved that these elements were key: 17 of 18 adult learning elements, 11 of 22 programming, 12 of 15 exercise, 7 of 8 upgrading exercises, 2 of 4 peer co-leader’s role, and all of the home visits, booster sessions, group leader’s role, and background and training of group leader elements. The top five key elements were: (1) use plain language, (2) develop trust, (3) engage people in what is meaningful and contextual for them, (4) train participants for cues in self-monitoring quality of exercises, and (5) group leader learns about exercises and understands how to progress them.

**Discussion:**

The Delphi consensus process suggested key elements related to *Stepping On* program delivery. These elements were considered essential to program effectiveness. Findings from this study laid the foundation for translation of *Stepping On* for broad US dissemination.

## Introduction

Falls among older adults can result in substantial morbidity and mortality, increased health-care visits and cost, and loss of independent living. In the past 15 years, significant progress has been made in identifying prevention strategies (1). However, while community-based programs have been shown to decrease the rate and incidence of falls, most have not been widely adopted by health-care or community service providers. Barriers to adoption and implementation may include lack of knowledge of key elements of the program, lack of expertise to train program providers, insufficient knowledge about the program target group, lack of funding, lack of a centralized registration process, and lack of a public awareness or marketing campaign (2–4). Due to dissemination factors in the environment at large, programs may need to be adapted to fit the local environment, but it is important to ensure that core elements do not change (5, 6). If core elements are not maintained, fidelity and program effectiveness may be lost. Manuals for community-based fall prevention programs ideally should provide sufficient information to allow organizations to understand what is a core element (i.e., an element that may *not* be adapted with implementation). However, the manual that is written for the testing phase of a new intervention may not provide sufficient information for community organizations to know what elements can be adapted, and what elements must be retained as essential for program effectiveness. Thus, before translating a program for widespread dissemination, it is essential to determine key elements (5, 6).

*Stepping On* is a group-based falls prevention program originally tested in Australia, where, in a randomized controlled trial, it was shown to reduce falls among community-dwelling elderly by 31% (7). For the randomized trial, Clemson et al. created a manual (8) outlining the conceptual basis of the program, background information on relevant topics, and step-by-step instructions on how to run each session. However, the randomized trial was not designed to elucidate the key elements of *Stepping On* that are essential for *effective* program delivery in practice, and the manual did not explain which elements may be adapted with community implementation and which may not.

*Stepping On* is a complex, multifactorial intervention conducted over seven 2-h weekly sessions with follow-up by a home visit and a 3-month booster session (1, 9). The content is based on current evidence-based knowledge of falls prevention strategies. The broad range of areas covered includes balance and strength training, home and community safety, and medication management. It is delivered as an educational program that uses adult learning principles (10), applies social-cognitive theory on influences of self-efficacy and skill mastery (11), and a decision-making model (12) to explore barriers and options for reducing risk and to facilitate the uptake of relevant strategies for participants. Various learning techniques are used including storytelling, reflection, and interpretation to help reframe ideas, brainstorming solutions to promote a sense of ownership, and the group process as a reflective and learning tool. Local experts [physical therapist (PT), pharmacist, low vision expert, traffic safety officer] are invited to present parts of the curriculum. Balance and strength exercise begins in session one, is practiced at home, and is progressed throughout the 7 weeks.

We introduced *Stepping On* to Wisconsin in 2006, and trained nine professionals as leaders through a 2-day training. At that point, the program had not been implemented previously in the US Training followed the Australian leader manual (13). The trainer was an RN who was experienced in multifactorial falls assessment (14) and was a master trainer for the Chronic Disease Self-Management program (15). Phone consultation with the program developer addressed questions. We found that the new Wisconsin leaders quickly modified the program in a number of ways: session order was changed; exercises were sometimes not taught; guest experts were sometimes omitted; the home visit following the 7-week session was omitted. According to stakeholders implementing the program, session order was changed and guest experts were omitted to increase ease of adoption and implementation. Leaders omitted exercises from a session when they ran short on time. The home visit was discontinued because of the high cost and burden, which could impede adoption. Key elements had not been elucidated by Dr. Clemson, and it was therefore impossible to know which modifications jeopardized program fidelity and effectiveness, and which did not.

In 2008, in response to a funding opportunity announcement (FOA) from the Centers for Disease Control and Prevention (CDC), we received funding to translate and package the *Stepping On* program for national dissemination, and conduct dissemination research (16). In the FOA, it was recommended that as a first step in translation, key elements of *Stepping On* be elucidated. Determining key elements in any intervention that is complex and multifaceted may be difficult. In some cases, the researchers/developers themselves define key elements (17). Subject matter expert opinion has been recommended as a valid method to determine which elements of a program are key (i.e., essential for program effectiveness), and which parts are not key (i.e., may be adapted if necessary without compromising program integrity) (18). Input from independent experts may strengthen and broaden decisions about key elements.

The purpose of this research study was to identify key elements of *Stepping On*, as suggested by content experts through use of a modified Delphi consensus, prior to packaging the program for widespread dissemination across the US.

## Materials and Methods

This study to identify content experts’ suggestions of key elements of *Stepping On* was the first step in a multi-step process to translate *Stepping On* for US audiences, guided by the Replicating Effective Programs (REP) framework (5). The complete translation process consisted of: (1) determination of key elements; (2) use of focus groups with adopters and end-users of the program to assess fit and acceptance of the program and provide input to program materials; (3) development of a draft program package based on key elements and stakeholder input; (4) implementation of the draft program and evaluation of implementation to ensure feasibility, fidelity, and positive outcomes; (5) revision of the program package based on findings from the pilot implementation; and (6) re-implementation of the program with evaluation, leading to final revisions.

We used a modified Delphi consensus technique, a widely used method to obtain unbiased expert consensus, to suggest key elements of the *Stepping On* program content (19). The modified Delphi technique allows for obtaining anonymous consensus, thereby mitigating bias related to differential status of group members. Developed in the 1950s, the Delphi technique has been used in varied subject areas as a systematic method for finding consensus (19, 20). In the Delphi method, experts begin by individually and anonymously answering an open-ended questionnaire about the content area. In subsequent rounds they then rate the importance of specific items. Questions are posed in a series of rounds until consensus is achieved or until it is obvious that future rounds will not provide additional information. At each round after the first, experts are provided feedback of anonymous comments from panelists in the round before. In the modified Delphi technique, the panelists begin with a set of items to rate for importance, rather than with an open-ended questionnaire. The beginning items are selected by the study team drawing from various sources including literature review and interviews with content experts (21). The primary advantage of using this modification is that it typically improves the initial response rate (21).

The study team who convened the Delphi panel and developed the questions for the panel consisted of: the *Stepping On* program creator (LC); a geriatrician with 15 years clinical and research experience in falls prevention and community-based research (JM); a nurse with 4 years of experience implementing community-based falls and chronic disease self-management interventions (SC); a PT with 10 years clinical and research experience in falls prevention (TS); the CDC’s program officer (KM); and three public health researchers with experience in injury prevention. Two of the study team members had prior experience with use of the modified Delphi technique.

### Selection of the Delphi Panelists

The study team identified a list of potential experts to serve on the Delphi panel. The team defined potential experts as individuals who had expertise related to the concepts, activities, and subject matter of *Stepping On*. This included experts in the areas of falls prevention, exercise, and self-efficacy-based interventions, as these were considered by the program developer as primary constructs of *Stepping On*. A list of subject matter experts from the US, Canada, and Australia was generated by the study team, based on study team members’ knowledge of the literature. The list included public health researchers and health professionals [PTs, occupational therapists (OTs), geriatricians] who were experts in falls prevention. The list also included exercise scientists with expertise in older adult exercise programming, researchers with expertise in self-efficacy-based interventions, and some of the early Australian leaders of *Stepping On*, based on their experience with implementing the program. We chose to include both independent experts in the field as well as *Stepping On* leaders, to mitigate potential bias from using either group alone. For example, program leaders could define key elements based on what they enjoyed doing in the workshop, what they found to be helpful, what they were skilled at, etc.

The letter of invitation informed experts that the Delphi study was being done as part of a larger study funded by the CDC to translate the Australian-based *Stepping On* into a US community program. They were informed that the specific aims of the grant included gathering information from content experts about key elements of *Stepping On* through the Delphi process, testing and evaluating implementation of *Stepping On* in community settings, and producing a final package for broad dissemination and use nationwide. All experts were informed that participation as a panelist would involve reviewing the original research article on *Stepping On* and the leader manual, and then participating in a Delphi panel of up to three rounds. Experts were informed that anonymity would be maintained by use of anonymous SurveyMonkey™, and that informed consent was not required per the University of Wisconsin Institutional Review Board.

Forty-four experts were invited to participate in the Delphi panel by the study team *via* email in waves to ensure that the panel would consist of equal numbers of PTs, OTs, geriatricians, exercise scientists, public health researchers, and Australian *Stepping On* leaders. For example, if the first PT refused, then the second PT on the list was invited, and so on. Table 1 shows the background of the 19 experts who agreed to serve as panelists in the Delphi consensus process.

**Table 1:** **Professional backgrounds of Delphi panelists**.

Professional backgrounds	N	%
Occupational therapist^a^	4	24
Physical therapist	3	18
Geriatrician	3	18
Epidemiologist	1	6
Research psychologist^b^	1	6
Public health/exercise scientist	1	6
Gerontologist/exercise physiologist	1	6
Kinesiology professor	1	6
Public health professional	1	6
Community fitness leader^c^	1	6

*^a^Two had conducted Stepping On; all had expertise with self-efficacy-based interventions*.

*^b^Had expertise with self-efficacy-based interventions*.

*^c^Had conducted Stepping On*.

### Development of Questions for Round One of the Delphi Panel

The study team developed the beginning set of questions for the modified Delphi panel. To do so, first each study team member reviewed the existing *Stepping On* Leader manual. The study team then met three times as a group by phone over 2 months to determine the domains into which the *Stepping On* program content and process should be organized. Some domains were suggested by the Australian program developer and others by other study team members, based on questions that arose from their experience with program implementation in Wisconsin. The final set of 10 domains was determined by consensus among study team members. The program developer suggested the domains of: adult learning, program parts, exercise, home visit, and booster session. Other study team members suggested the domains of: training and background of group leader, role of peer co-leader, and manual, based on questions that had arisen from their experiences with program implementation in Wisconsin. After discussion, the study team added the domain of “role of group leader,” to categorize elements related to group facilitation.

After determining domains, through four meetings by phone over 3 months, the study team generated questions within each domain regarding potential elements of program effectiveness. The final round one questionnaire consisted of 112 items over the 10 domains: adult learning (18 items), program parts (21 items), exercise (23 items), qualifications of invited experts to introduce exercise (4 items), home visit (13 items), booster session (4 items), role of group leader (12 items), training and background of group leader (10 items), role of peer co-leader (4 items), and manual (3 items).

Most questions in round one described an element used in the *Stepping On* program, and asked: “How important is this element to the program’s effectiveness?” For example, for the domain of “adult learning,” the survey stated “The manual describes a number of elements that are used in adult learning to help engage in homework and class participation.” One element provided under that section was “Invite feedback,” followed by question “How important is this element to the program’s effectiveness?” The panelists were asked to rate importance on a scale of 1 = “Not important at all”; 2 = “Probably not important”; 3 = “Possibly important”; 4 = “Very Important”; and 5 = “Essential.” “Essential to the program’s effectiveness” was defined in the cover letter as indicating “an element whose absence would reduce the program’s effectiveness” in reducing falls among program participants. The 5-point scale of importance has been used to elucidate key components of yoga interventions for musculoskeletal conditions (22).

Some questions used a scale of 1 = “Definitely not”; 2 = Probably Not; 3 = Unsure; 4 = Probably; and 5 = Definitely. Examples of these questions included: “Handouts are provided in the *Stepping On* manual. Do you think the handouts express the key things that are important for adult learners?” and “Do you think the number of exercises provided in the manual is sufficient?” These questions also asked about types of professionals that could serve as the invited expert instead of a PT to introduce the exercise concepts (e.g., OT, PT assistant, therapy OT, fitness expert). We provided no further definitions of the points on the scale. All questions gave a choice of “unable to answer.” At the beginning of the survey panelists were instructed “Please check a response for every item of each question. You are required to answer each item before moving on, so check ‘unable to answer’ if you do not feel you can answer the item.” Panelists were asked to provide any comments they wished about any of their responses, and a space for comments was provided at the end of each domain of questions (e.g., adult learning elements, programming, exercise, upgrading exercise, choices of professionals to serve as invited expert to introduce exercise concepts). The round one survey was piloted with three Wisconsin *Stepping On* leaders to ensure clarity of questions and response categories.

### Selection of Questions for Rounds Two and Three

For computerized versions of the Delphi process, it is essential to have a moderator whose role is to synthesize information from each round and make decisions about what should be provided back to the group (23). The study team served the function of moderator, reviewing scores and comments from round one questions and then meeting *via* telephone conference to develop round two questions based on round one results, then doing the same for round three, based on round two results. All scores and comments from each round were tabulated and provided verbatim to the study team. The study team examined all items that did not achieve consensus in round one to determine which should be advanced to round two. To reduce respondent burden, the study team only selected items for round two that had significant controversy (i.e., a very broad distribution of responses across the 5-point scale) in round one. For questions being advanced to round two, the study team repeated or rephrased the round one question in the round two survey, and provided the comments and responses that the panelists had given in round one (e.g., the percent of panelists scoring essential, very important, possibly important, probably not important, and not important at all), Then the panelists were asked to re-score the question, using the same scale as in round one, and provide a rationale for their score. In round three, the same occurred, with the study team giving the range of responses and comments from round two.

In some cases, a panelist’s comment from the previous round suggested that the question was unclear. In these cases, the study team clarified the question in round two or three. For example, in round one panelists were asked whether a “group size of 10–14 participants” was essential. One panelist commented “With respect to group size a smaller group of 8–10 members may be more manageable for the facilitators (and productive) when there are more ‘higher need’ individuals in the group.” This led the study team to clarify the wording of the question in round two to ask whether a “group size *limited to* 10–14 participants” was essential. As another example, in round one, the question was asked: “Should the manual have more information about maintaining safety with the community mobility session?” In round two, this was reworded for clarity as “Should the manual have more information about preventing falls and injuries from occurring while conducting the community mobility session?”

Some comments suggested ways of viewing elements that the study team had not considered. These comments were provided back verbatim to the panelists in round two or three. For example, one panelist in round one commented that anyone who has experience with exercise training and can effectively lead an exercise session would be qualified to serve as the invited expert to introduce exercise concepts in *Stepping On*. We added this as a question in round two, asking panelists “Would anyone who has experience with exercise training and effectively lead an exercise session be qualified to introduce exercise concepts?” using the scale 5 = “Definitely” to 1 = “Definitely not.” Panelists were asked to provide a rationale for each of their scores.

The Delphi process consisted of three rounds, with 3 months between each round. Round one survey required 30–40 min to complete; round two required 15–30 min; and round three required 5 min. All 19 panelists completed the first round. Two panelists (11%) did not complete rounds two and three. Because all responses were anonymous, we could not identify which panelists did not complete rounds two and three and why. The University of Wisconsin Health Sciences Institutional Review Office reviewed the Delphi Consensus protocol, which was determined to be exempt.

### Data Analysis

Data was exported from SurveyMonkey™ to Excel for analysis. Descriptive statistics were used to provide frequencies of responses for each question. The primary outcome for each item was consensus that it was key element, consensus that it was not a key element, or no consensus.

Consensus in round one was defined as 70% of the panelists scoring in one category, with the categories of “Essential” and “Very important” combined. In round two, consensus that an item was a key element was defined as 80% scoring in “Essential,” “Very important,” or “Possibly important,” as long as the “Possibly important” category did not equal or exceed 50%. Consensus that an item was not a key element was defined as >80% scoring in the “Possibly important,” “Probably not important,” or “Not important at all” categories, if the “Possibly important” category did not equal or exceed 50%. In determining the criteria for round two, the study team made the decision to combine the “possibly important” group with the other “important” options for the items where there were a minority of responses in the “Possibly important” category, taking into consideration that there had been little movement in that category compared to round one. The study team chose this approach because it conserved items as essential when there was a majority distribution of responses among the three categories.

A Rasch analysis of item fit (Winsteps Ver 3.72) was conducted on round one responses to explore the degree of diversity and/or homogeneity of responses of the panel. Rasch analysis allows responses from individuals to be tested against response patterns predicted by the model. The pattern expected by the model is a probabilistic form of Guttman structure, which is a deterministic model that has a strict hierarchical ordering of items (24). This allowed us to examine responses to see if, as expected, those items that were less likely to be endorsed by experts would be rated of lower importance, and *vice versa* (25). Rasch also enables a way to empirically test if the respondents are able to differentiate within the questions and rating scales (e.g. infit outfit statistics). Infit statistics give more weight to persons and items in the middle of the range. The unweighted outfit statistic is more sensitive to the presence of outliers (25, 26). Mean square fit statistics are considered at best fit with the Rasch model when centered at one, with a range of 0.60–1.49 with concomitant *Z* standardized scores between −0.2 and.2 and a point-measure correlation with the Rasch logit measure with the responses (25).

## Results

### Round One

Table 1 shows the professional backgrounds of the 19 Delphi panelists and whether they had experience leading the *Stepping On* program. Three panelists had previously implemented *Stepping On*. Five panelists had expertise with self-efficacy based interventions.

The round one survey consisted of 112 items over 10 domains. For 88 items, panelists were asked to rate the importance of each item for the *Stepping On* program’s effectiveness in reducing falls, using a rating scale from 1 = not important at all, to 5 = essential. Twenty-four questions asked about items related to program implementation using a rating scale from 1 = definitely not, to 5 = definitely. The number of panelists completing each individual question ranged from 17 to 19 with a mode of 18. At the end of round one, 69 items reached consensus. Table 2 shows items reaching consensus in round one.

**Table 2:** **Summary of items with consensus as key elements, % agreement as essential or very important, and round in which consensus occurred**.

Element	% Agreement	Round in which consensus^a^ occurred
**Adult learning elements considered essential or very important**		
Plain language	100	1
Develop trust	100	1
Engage people in what is meaningful and contextual for them	100	1
Introductions	94	1
Use optimism and positive talk	94	1
Link strategies and skills to personal goals	94	1
Facilitate engagement of all members of group	94	1
Environment	90	1
Invite feedback	89	1
Keep group focused	89	1
Use story	89	1
Help break down solutions into simple steps	89	1
Use prevention framework	82	1
Slow pace	79	1
Use a variety of medium to support learning styles	78	1
Invite group suggest topics	72	1
Include discussion of last week’s topics	72	1
**Program aspects considered essential or very important**		
Final group evaluation in the last session	95	1
Objectives reviewed with group	89	1
Invited experts prepped ahead of time by leader	89	1
Class leader reviews key messages from invited experts	89	1
The prior week’s homework is reviewed each session	84	1
Medication record card, with group discussion	84	1
Snacks and beverages	84	1
Homework is assigned each session	79	1
Topic handouts	74	1
Apple game (i.e., knowledge quiz) with group discussion	74	1
Group size of 10–14 participants	83	2
**Exercise elements considered essential or very important**		
Train participants in cues for self-monitoring quality of exercises	100	1
Group leader learns about exercises and understands how to progress them	100	1
Group leader links exercises to preventing falls	100	1
Group leader shows where to buy or obtain weights, and how to put on ankle weights	95	1
Introduce the exercises in the first session	89	1
Group leader has weights available at the class for participants to borrow	84	1
Each session has some exercise	83	2
Introduce the concept of advancing exercises at the first session	77	1
Group leader encourages snacking	72	1
Group leader collects exercise homework	72	1
All exercises in the manual are taught	62	2
Exercises are limited to only those included in the manual	33	1
**Upgrading exercise elements considered essential or very important**		
The group leader learns about exercises and how to upgrade them	100	1
The group leader believing that upgrading exercise is important	96	1
The group leader encouraging participants to advance exercises, as able, throughout the sessions	94	1
Teaching the participants the importance of challenge to balance (session one)	89	1
The group leader having strong self-efficacy that he/she can safely progress exercises	89	1
The group leader encouraging participants to advance to not holding on during exercise, as able, throughout the sessions	88	1
The group leader encouraging the use of weights, as able, throughout the sessions	78	1
**Home visit elements considered essential or very important**		
Assistance with follow-through of falls prevention strategies and activities	100	1
Reinforcement of those falls prevention activities that have been accomplished	100	1
Support and, if necessary, assistance with putting into practice the safety strategies they have learned related to home and community environment	95	1
Supplementation of participant’s assessments of falls hazards in and about the home	77	1
Assistance with home adaptations and modifications, if required	78	1
Assistance with referral to support services (upon request)	89	1
How important is it that the session occur in the home (as opposed to over the phone)?	89	1
**Booster session elements considered essential or very important**		
Objective of reviewing exercise barriers and facilitators	95	1
How important is the booster session?	94	1
Objective of reviewing changes that have been put in practice	88	1
The timing of the booster session is three months	59	2
**Group leader’s role: elements considered essential or very important**		
Leader facilitates increased sense of ownership by participants	100	1
Leader inquires about and accommodates needs related to vision or hearing impairment	95	1
Leader debriefs with the co-leader after each class	95	1
Leader is skilled at interpreting themes and reframing ideas	89	1
Leader provides monitoring and feedback to invited expert regarding getting across key messages, using relevant examples, using group process, using plain language	89	1
Leader understands the concept of “target the behavior for change”	84	1
Leader provides instruction to key expert before expert comes	84	1
Leader is skilled at prompting “story telling”	83	1
Leader is skilled at “story telling”	78	1
Leader is skilled in using the decision making framework	78	1
Leader calls people who miss a session	78	1
When facilitating, leader presents self as equal with participants in the group	56	1
**Background and training of the group leader: elements considered essential or very important**		
The group leader has the ability to work with seniors (i.e., experience, understanding their needs)^b^	100	2
The group leader has a good knowledge of exercise	94	1
The group leader has a good knowledge of falls prevention topics	94	1
The group leader has previous experience with facilitating adult groups	88	1
**Background of group leader: besides a physical therapist (PT), RN, or occupational therapist (OT), professions that could definitely or probably fulfill the role of group leader**		
Retired PT, OT	83	2
Social worker	82	3
Physical therapy assistant (PTA)	76	2
Health educator	76	2
Fitness expert	76	2
LPN	64	2
**Elements of peer co-leader role considered essential or very important**		
Prompting questions	71	1
Role modeling how to be an active participant in the class	70	1
**Qualifications of invited expert who introduces exercise (definitely or probably acceptable)**		
Fitness expert	94	2
PTA	89	2
Health professional with exercise training or exercise experience with older adults	88	2
OT	76	2

*^a^Positive consensus for round one was indicated by 70% response in a category, with “essential” and “very important” categories combined. Positive consensus for round two was indicated by 80% response in “essential,” “very important,” and “possibly important” categories combined, as long as the “possibly important” category did not approach or exceed 50%. For the scale of Definitely to Definitely Not, positive consensus was indicated by 80% response in “definitely,” “probably,” or “unsure” categories, as long as the unsure category did not approach or exceed 50%*.

*^b^This question was only asked in round two*.

The Rasch analysis of the 19 panelists using fit statistics shows that there was a reasonable and sufficient range of diverse perspectives (Infit MnSQ 1.01, *Z* score −0.1, Outfit MnSQ 0.96, *Z* score −0.2 with a separation of 4.89). Only one panelist was “misfitting” (Infit MnSQ 1.63, *Z* score 4.2, Outfit MNSQ 1.69, *Z* score 4.2) showing that they assessed items very differently than the other panelists. Another panelist (Point-measure correlation = 0.25) contributed the least, responding to most items as “essential” and not responding to others.

### Round Two

The study team examined all items that did not achieve consensus in round one to determine which items should advance to round two. Table 3 shows items that were not advanced to round two. The round two survey consisted of 26 items. Twenty round two questions used the definitely/probably scale. These were questions about who could fulfill the role of invited expert or group leader, and whether or not adaptations could be made to handouts, exercises, or other programmatic elements. Six questions used the essential/very important scale; these related to whether an item was a key element.

**Table 3:** **Elements not achieving consensus as key to *Stepping On***.

Element	Direction of responses	Consensus achieved or not	Round
**Adult learning**			
Use breaks and asides	Important	No	1^a^
**Program**			
Objectives handouts	Important	No	1^a^
Topic handouts provided after brainstorming	Important	No	1^a^
Sessions presented in same order as in manual	Possibly important	No	2
Apple game without group discussion, session five	Not important	No	1^a^
Former participant provides reflections, session five	Important	No	1^a^
Shopping list is used to determine group wants, sessions one and two	Important	No	1^a^
Medication record card without group discussion	Not important	No	1^a^
Invited experts without prepping ahead of time by leader	Important	No	1^a^
Display table	Important	No	1^a^
The group leader should encourage attendance at local exercise venue only if they offer balance exercises	Probably not	No	1^a^
Peer co-leader	Possibly important	No	2^b^
**Exercises**			
Introducing the concepts of weights in the second session	Important	No	1^a^
Exercises should be limited to only those included in the manual	Not important	No	1^a^
The program should provide alternative exercises	Definitely	No	2
**Activities of home visit being accomplished via phone**			
Assistance with follow-through of falls prevention strategies and activities	Probably	No	1^a^
Reinforcement of falls prevention activities that have been accomplished	Probably	No	1^a^
Provide support and assistance if necessary with putting into practice the safety strategies they have learned related to home and community environment	Probably	No	1^a^
Supplementation of participant’s assessments of falls hazards in and about the home	Probably not	No	1^a^
Assistance with home adaptations and modifications if required	Probably not	No	1^a^
Assistance with referral to support services upon request	Probably	No	1^a^
**Peer co-leader role**			
Peer review of facilitation skills	Important	No	1^a^
Leading parts of sessions	Important	No	1^a^
**Background of person who could fulfill role of group leader**			
Nutritionist	Probably	No	3
Director of a senior center	Probably	No	3
Student in health profession	Unsure	No	2

*^a^Not asked in round two. Items were not asked in round two to reduce respondent burden. The study team felt that for Program and Exercise items, responses from round one provided enough directionality of importance to guide implementation nationally. For Activities of home visit being accomplished via phone, the study team subsequently conducted a mixed-methods study to answer this question. For the Peer co-leader role, the questions were re-framed into one round two question about the importance of the peer co-leader in general. For the background of person who could fulfill role of group leader, the study team subsequently conducted a mixed-methods study to answer this question*.

*^b^Only asked in round two*.

Seventeen panelists participated in round two. Table 2 shows the 25 items that reached consensus at the end of round two. Only one item did not achieve consensus.

### Round Three

The round three survey consisted of the one item that did not achieve consensus in round two. This item dealt with which professions, in addition to OTs and PTs, could fulfill the role of *Stepping On* leader. For round three, the study team re-framed the question from round two based on qualitative responses that panelists had provided in rounds one and two. For example, some panelists said it was more important for future *Stepping On* leaders to have skills in group facilitation and prior experience working with seniors, than to have a medical background.

Therefore, the new question was: “Currently, we have a two-and-a-half-day training for *Stepping On* leaders that includes training on fall prevention content as in the *Stepping On* manual. For this question, please assume all potential leaders have skills in group facilitation and knowledge of adult learning principles as well as having prior experience working with seniors. Would professionals such as social workers, nutritionists, or directors of senior centers be acceptable to fulfill the role of *Stepping On* leader if they take the two and a half day training and can demonstrate mastery of the fall prevention content in the manual?” All scores and responses from rounds one and two that related to this item were given back to the panel. Seventeen panelists participated in round three.

### Summary of All Rounds

Table 2 summarizes items that achieved consensus as key elements, the percent of respondents agreeing with the item being key, and the round in which consensus was achieved. Consensus was achieved for 17 of 18 (94%) elements of adult learning, 11 of 22 (50%) programming elements, 12 of 15 (80%) exercise elements, 7 of 8 (88%) elements related to upgrading exercises, all 7 home visit elements, all 4 booster session elements, all 12 elements related to group leader’s role, all 5 elements related to background and training of group leader, and 2 of 4 elements related to the peer co-leader’s role.

The top 10 items achieving consensus (100% agreement) were (1) use plain language, (2) develop trust, (3) engage people in what is meaningful and contextual for them, (4) train participants for cues in self-monitoring quality of exercises, (5) group leader learns about exercises and understands how to progress them, (6) group leader links exercises to preventing falls, (7) the group leader learns about how to upgrade exercises, (8) the home visit provides assistance with follow-through of falls prevention strategies and activities, (9) reinforces those falls prevention activities that have been accomplished, and (10) the leader facilitates increased sense of ownership by participants.

Table 3 shows areas of no consensus, the directionality of responses for those areas, and the last round in which the question was asked. Programmatic aspects without group discussion (e.g., knowledge quizzes, handouts) were not considered key, but, as shown in Table 2, were considered key when done with group discussion. Invited experts, without having the group leader prep them ahead of time, were not considered key, but were reconsidered as key when prepped ahead of time. This suggests that the importance of certain activities depended on how the activities were implemented.

Although panelists did not reach consensus in round one, most felt that the number of exercises provided in the manual was sufficient. Most also felt that alternative exercises should be provided. Because of a wide variety of comments, round two further clarified questions related to exercises. There was no consensus about whether alternative exercises should be provided. Comments revealed that there should be ways to modify the exercises to accommodate those with physical limitations, but that the invited expert should be able to make modifications from the exercises provided. Comments included: “Older adults will ‘tell’ you how he/she would need to modify the activity. The lead instructor needs to check that the modification and progression is safe”; “Need to be sure the older adult is performing the activity within their functional capability. Functional capability is more important than doing the activity ‘perfectly’”; “Activities may need to be adapted according to the health status of the older adult”; “Adapt to individual need”; “The use of a physiotherapist to introduce the exercises means that individuals can ask specific questions about each exercise and if they experience problems, what alternatives there are”; “Some people may need alternative exercise”; “Sometimes important to adapt to unique personal or environmental settings”. Thus, panelists espoused modification of exercises for frailer participants.

The Delphi panelists provided a number of suggestions regarding the participant handouts. Comments included “Update the home safety assessment form—seems written for a third party; substitute with a form that is easier to use”; “Review handouts for reading level and font size”; “Home hazard screening checklist—add suggestions for outside of the home”; “Check reading comprehension and cultural sensitivity of all handouts.”

The home visit was a key element. Each of the six activities of the home visit was considered key. There was no consensus that any of these activities could be accomplished by phone.

There was consensus regarding the skill set the leader would need to bring to the role: experience with adult group facilitation, knowledge of falls prevention and exercise, and experience with seniors. However, there was less consensus regarding type of profession of the person who could fulfill the role of leader. Round three asked if a professional such as a social worker, nutritionist, or director of a senior center would be acceptable to fulfill the role of leader, provided that the person had skills in group facilitation, knowledge of adult learning principles, and prior experience working with seniors. It presumed that the professional would take a two and a half day training at the end of which he/she would demonstrate mastery of falls content. Round three showed consensus that a social worker could fulfill the role of leader, but there was no consensus that a senior center director or nutritionist could. A divergence emerged in comments. Three panelists (18%) (PT, OT, and geriatrician) said none of the above categories could serve as leader. Among these three, two stated only PTs or OTs could lead; one provided no comment. Eleven panelists stated that any professional expertise would be acceptable, provided they met the criteria posed in the question. All respondents with prior experience leading *Stepping On* (two OTs, one community worker) felt any professional would be acceptable if they met the criteria posed.

Panelists who answered that any professional could “probably” or “definitely” serve as leaders commented that the elements for a successful leader were prior experience with older adults, motivation to run the program (i.e., person chooses to do it rather than is chosen by a superior), skills in group facilitation, knowledge of adult learning principles, mastery of manual content, including falls prevention content, and awareness of basic safety principles. Panelists stated that to ensure above criteria are met, there should be a screening process before training, a test of mastery of content and safety principles after training, a demonstration of group facilitation skills, and a monitoring process after training to ensure the program proceeds with fidelity to key elements.

## Discussion

In this study, use of the modified Delphi Technique elicited expert consensus suggesting key elements of *Stepping On*. Panelists reached consensus on most items in round one. Key areas of agreement centered on conceptual underpinnings of the program, roles of the leader, exercise elements, and other program elements. Our findings supported that the conceptual underpinning of the program and the group process were integral to learning and uptake of prevention strategies and that leaders required skills in knowledge of falls prevention evidence, group work, and principles of adult learning, decision making, and self-efficacy.

Findings from the Delphi study guided development of the US program package in several ways. First, findings informed how new leaders are selected, trained, and coached. *Stepping On* leaders are expected to meet the criteria established by the Delphi consensus. Second, in the program manual, key elements are signified with a “key” icon, and the manual’s toolkit provides a list of all key elements suggested by the Delphi panel. Third, findings led to development of a new 3-day leader training, which focused on didactics and practice to achieve competency in implementing key elements in practice. Before being certified as a *Stepping On* leader, trainees must pass a knowledge test of key elements and a competency test to show they are able to use the key elements in practice. Fourth, after certification, new leaders are monitored for fidelity as they facilitate one session of their first *Stepping On* workshop, and are coached afterward to improve performance. Fifth, findings informed the development of the *Stepping On* Site Implementation Guide that is used nationally to help organizations prepare to adopt the program with fidelity to key elements (27). Sixth, the findings guided an understanding of what elements are not essential. These were elements where the Delphi panel did not achieve consensus. During training, leaders now are taught that elements that are non-essential may be adapted for their setting. For example, the order in which invited experts such as the pharmacists and optometrist attend sessions is not essential. Content of sessions may be rearranged so the pharmacist can come to a later session if he/she cannot come to the one originally specified in the manual. Such site-specific adaptation of non-essential elements facilitates adoption and implementation (5, 6).

Translation of a program for broad dissemination cannot be based solely on expert consensus of key elements; it also requires input from stakeholders who have implemented the program, in order to ensure broad successful adaptation. We engaged in a number of steps to ensure that the US *Stepping On* translation incorporated stakeholder input. Following the approach outlined in the CDC’s FOA, immediately after the Delphi consensus, we conducted focus groups and surveys of former participants, leaders, and guest experts of *Stepping On*. Two focus groups of former participants (one rural and one urban) provided information about barriers to participation and completion of *Stepping On* homework, about readability and understandability of the handouts, and what worked and what did not in the workshop. Two focus groups of leaders provided information about barriers to hosting and leading the workshop, and what worked and did not with leading the workshop. In addition, surveys gathered data from leaders who had received training in *Stepping On* but had not yet led a class (to evaluate barriers to class start-up); from former participants who had not completed the program (i.e., attended fewer than five of seven sessions; to evaluate barriers to completing the class); and from invited experts (to evaluate barriers and facilitators to participating as an invited expert, and barriers and facilitators to implementing with fidelity). The findings from focus groups and surveys provided essential information on how to adapt the program to meet setting-specific needs. The findings enabled us to provide a menu of options to leaders and sponsoring organizations so they could adapt delivery for their site, while still maintaining fidelity to the suggested key elements. For example, the need to modify exercises for frailer participants was confirmed by the findings of the survey of PT invited experts. As a result, in the draft leader manual we provided exercise modifications that the invited PT could use. Last, findings allowed us to modify handouts to maximize their acceptability and usability for participants.

Guided by the CDC’s FOA, we developed a draft program package that emphasized the key elements suggested by the Delphi consensus and incorporated the insights from focus groups and surveys of *Stepping On* participants, leaders, and invited experts. All handouts were checked with Microsoft Word diagnostics to ensure third grade reading level. We trained a new leader and co-leader, who implemented the draft program package in a senior retirement community. We monitored program implementation for fidelity, identified lapses in fidelity, and further revised the package to ensure fidelity with future implementation (28). Lastly, we retrained the leader, who implemented the program a second time. Fidelity monitoring showed improved fidelity with the second implementation. We then made final revisions to the draft program package based on feedback from participants, leaders, and invited experts. These steps followed the REP framework, which was developed and used by the CDC to operationalize the sequence of actions needed to prepare community-based HIV interventions for broad dissemination (5). Supporting the validity of utilizing both the Delphi consensus and information from the field to inform the translation process, subsequent evaluation of the US *Stepping On* program with over 2,300 participants was associated with over 30% reduction in falls during the six months after the program compared to 6 months before, consistent with the effectiveness found in the original *Stepping On* study (29, 30).

### Items with Consensus

All panelists rated adult learning theory as “essential” out of the three major programmatic constructs of *Stepping On*. These key programmatic aspects perceive the participant as having an active role in the process, that the program engages people in what is meaningful and contextual for them, and supports the leader’s role in facilitating a sense of ownership of strategies and solutions. Aspects that incorporate principles of self-efficacy were rated overall as “highly important,” such as mastery of specific skills, the use of optimism and positive affirmation of accomplishments, and the power of role models through storytelling. The role of decision making also achieved consensus as a key element. This was assessed by one item related to the use of the preventive framework, that is, the prompts used by the leader throughout the program to encourage decision and action. Other strongly supported items focus on elements vital to uptake and on embedding preventive strategies into routine practice and maintenance over the long term.

Teaching the home-based balance and strength exercises was generally perceived of prime importance with all panelists endorsing, as essential, the leader beliefs and skills in upgrading, their ability to teach how the exercises are relevant to falls prevention, and their ability to give participants strategies for self-monitoring. Program aspects around home environmental safety and medication management were considered key if they included participant discussion along with practical learning opportunities.

One highly rated element was for supporting the *Stepping On* participants in reflecting on their accomplishments in the final session of the program. Delphi panelists endorsed the home visit at the conclusion of the seven sessions and a 3-month follow-up booster session as essential for reinforcing accomplishments, and for reviewing enablers and barriers to the exercises. This is consistent with the evidence that supports that follow-up can improve exercise maintenance and assist in coping with relapse (31–33).

### Items without Consensus or Where Consensus Depended on Context

It was important to identify areas that lacked consensus, or where consensus depended on the context with which the element would be implemented in *Stepping On*. For example, when the apple game or medication card were used without group discussion, they were not important elements to *Stepping On*’s success. When used with group discussion, they were essential. Similarly, invited experts without prepping ahead of the time were not considered important, but with prepping ahead of time, were considered essential. Some programmatic and exercise-related elements (for example providing handouts of the objectives for each session) did not reach the threshold of “essential” although the directionality of scoring favored their importance. These elements were added to the *Stepping On* national package as being “strongly advised.”

We identified numerous areas of controversy, which are important as they affect potential cost, reach, uptake, and maintenance. For example, the relative importance of the home visit versus a phone call could not be determined through the Delphi process. We subsequently evaluated this question as part of the testing of the US program package. Results of this evaluation are reported elsewhere in this journal. In addition, while some consensus was reached as to the kind of professional best suited to the leader’s role, consensus was not reached as to what type of professional would not be suited to this role. Of note, the three panelists who stated that professionals such as a senior center director or nutritionist could not fulfill the role of leader, were all health professionals. In contrast, panelists with prior experience leading *Stepping On* felt any professional would be acceptable if they had experience in group facilitation, knowledge of adult learning principles, and prior experience working with seniors, and if they received a two and half day training at the end of which they demonstrated mastery. It is an important question whether a non-professional (lay) leader, given the appropriate training and background of experience, can successfully conduct *Stepping On*. The Chronic Disease Self-Management Program, led by lay leaders, demonstrated success in decreasing hospitalizations and emergency room visits in a randomized trial (15, 34). The Matter of Balance intervention to decrease fear of falling used trained OTs (35) in its successful randomized trial, and now in adaptation, uses lay leaders (36). *Stepping On* leaders are required to have mastery of falls prevention content and of balance and strength exercises, and to apply adult learning principles, decision making theory, and other key concepts in addition to group work skills. It is unclear if the positive results with use of lay leaders in other self-efficacy based health promotion programs would translate to success with *Stepping On*. We undertook subsequent research to answer this question, examining fidelity of the program with implementation by leaders of different disciplines and backgrounds. We also examined outcomes associated with implementation of *Stepping On* with a home visit versus a phone call. Results of these studies are reported elsewhere in this journal.^1^

### Use of the Delphi Method

While the Delphi method has been used frequently in health care research, there are relatively few examples of its use to aid translation of community-based interventions from research into practice. Health care researchers have used it to determine best palliative care practices for older adults with dementia (37), prescribing indicators for UK general practice (38), potentially inappropriate medications for older adults (39), criteria for developing and validating a falls environmental checklist (40), and criteria for appraising the quality of patient decision aids (41), among others. This research demonstrates another important use for the Delphi consensus: to aid in understanding key elements of community-based interventions, as a first step to enable program translation from research into practice. It is important to note that the expertise of the study team with regard to *Stepping On* was critical to item selection for the Delphi study, which in turn was critical to the study’s success. It is also important to note that elucidation of key elements was only the first step in a process that also included stakeholder feedback, a necessary part of the translation process, as recommended in both the Consolidated Framework for Implementation Research (CFIR) and the REP framework (5, 6).

### Strengths and Limitations

This study has several strengths. First, panelists were internationally known experts in their fields and all gained knowledge of *Stepping On* through reading the leader manual and the original research article. Second, bias from overemphasis on any specific profession was minimized by ensuring that panelists were represented in equal numbers from the disciplines of PT, OT, geriatrics, exercise science, and self-efficacy/behavior change science. We included *Stepping On* leaders as well as non-leader experts in pertinent fields. Leaders may be biased by what they enjoyed or didn’t enjoy doing in *Stepping On*, and what they found easy or difficult to implement. To safeguard against potential bias from only including *Stepping On* leaders, we included non-leader experts in falls prevention content and group self-management process as well. Consistent with this approach, diversity in consensus panels has been noted to improve performance (19).

A possible limitation was that non-leaders may have had limited knowledge of how to facilitate the program. This was unlikely to be a significant limitation for several reasons. First, the non-leaders included three professionals (2 OT’s and a research psychologist) who had expertise in self-efficacy based interventions, who could, therefore, also speak to elements related to group facilitation and adult learning. Second, all panelists were asked to review the *Stepping On* leader manual, whose introduction explained in detail the program’s foundation on self-efficacy theory and adult learning principles. Third, the Delphi technique itself, which creates a forum for anonymous sharing of opinions and rationales over multiple rounds, creates an environment where all voices contribute equally to consensus formation. Fourth, the key elements suggested by panelists were consistent with the program’s theoretical foundations. A second limitation is that the heterogeneous nature of the Delphi panel may have limited consensus in some areas. Third, the Delphi panel was small and this may increase bias in responses, although Murphy et al. argue that consensus panels above 12 participants may show diminishing returns (19). Lastly, the panel was not asked to reflect on key elements related to dissemination to communities of color. Future research would be beneficial to determine the applicability of key elements or the need for adaptation for African American, tribal, or Hispanic populations.

## Conclusion

In summary, this Delphi consensus suggested key elements related to *Stepping On* program delivery across 10 domains ranging from leader background and training, to adult learning elements, programmatic aspects, and exercise elements. These elements and domains were seen as essential to program effectiveness in reducing falls. The Delphi panel’s consensus served as the foundation for development of the US program package, with subsequent research demonstrating its effectiveness in reducing falls. The US *Stepping On* program package is administered now by the Wisconsin Institute for Healthy Aging (WIHA; https://wihealthyaging.org) which, through agreement with the Australian program’s developer, trains and certifies Leaders and Master Trainers, distributes materials and issues licenses to organizations to implement *Stepping On*, and provides pre and post training support to leaders and adopting organizations, all with the goal of maintaining fidelity to the key elements suggested by the Delphi panel.

## Author Notes

Jane Mahoney, M.D., is board certified in geriatrics and internal medicine. She is a Professor of Geriatrics in the University of Wisconsin School of Medicine and Public Health. She also serves as Executive Director of Wisconsin Institute for Healthy Aging, a non-profit organization that disseminates evidence-based prevention programs for older adults. She is Principal Investigator of the Community-Academic Aging Research Network, a NIA-funded initiative to support research collaboration between University of Wisconsin researchers and community partners from Wisconsin’s Aging Network. Dr. Mahoney has received funding from the American Physical Therapy Foundation, CDC, the NIA, and the State of Wisconsin for epidemiologic and clinical research on falls. She has studied risk factors for falls after hospitalization, clinical trials of community-based multifactorial falls interventions, and dissemination research on the *Stepping On* falls prevention program. She is currently working with University of Wisconsin’s Active Aging Research Center to help develop internet-based technologies to help older adults reduce falls and to maintain independence.

This manuscript was presented in part at the 2012 Gerontological Society of America Annual Meeting, San Diego, CA, USA.

## Author Contributions

JM was responsible for conceptualizing the theoretical and empirical formulations of each research project, literature review, study protocol and design, and for collecting, analyzing, and interpreting data and manuscript preparation. KM is a co-author and offered substantive intellectual input and expertise during each phase of the research formulation and manuscript preparation, and provided feedback on all drafts. TS was part of the original study group that developed and analyzed the Delphi data, feedback on the presentation at the GSA in 2012, and provided feedback on draft versions of the paper. I have read and have approved the final version.

## Disclaimer

The findings and conclusions in this manuscript are those of the authors and do not necessarily represent the views of the Centers for Disease Control and Prevention/the Agency for Toxic Substances and Disease Registry.

## Conflict of Interest Statement

JM and LC are Co-Authors on the *Stepping On* Leader Manual, Third North American Edition, Freiburg Press, Cedar Falls, IA; 2011. The remaining co-authors declare that the research was conducted in the absence of any commercial or financial relationships that could be construed as a potential conflict of interest.

## Footnotes

^1^Schlotthauer A, Mahoney JE, Christainsen A, Gobel VL, Layde P, Lecey V, et al. Implementation of Stepping On in three communities. *Front Public Health* (submitted).

## Abbreviations

CDC, Centers for Disease Control and Prevention; MnSQ, mean square; PT, physical therapist or physical therapy; OT, occupational therapist or occupational therapy; RN, registered nurse; PTA, physical therapy assistant; LPN, licensed practical nurse.
